# Development of primary care assessment tool–adult version in Tibet: implication for low- and middle-income countries

**DOI:** 10.1017/S1463423619000239

**Published:** 2019-07-01

**Authors:** Wenhua Wang, Jeannie Haggerty

**Affiliations:** Department of Family Medicine, McGill University, Montreal, Canada

**Keywords:** confirmatory factor analysis, item response theory, patient experience, Primary Care Assessment Tool, primary health care, psychometric analysis

## Abstract

**Aim:**

To conduct advanced psychometric analysis of Primary Care Assessment Tool (PCAT) in Tibet and identify avenues for metric performance improvement.

**Background:**

Measuring progress toward high-performing primary health care can contribute to the achievement of sustainable development goals. The adult version of PCAT is an instrument for measuring patient experience, with key elements of primary care. It has been extensively used and validated internationally. However, only little information is available regarding its psychometric properties obtained based on advanced analysis.

**Methods:**

We used data collected from 1386 primary care users in two prefectures in Tibet. First, iterative confirmatory factor analysis examined the fit of the primary care construct in the original tool. Then item response theory analysis evaluated how well the questions and individual response options perform at different levels of patient experience. Finally, multiple logistic regression modeling examined the predicative validity of primary care domains against patient satisfaction.

**Findings:**

A best final structure for the PCAT-Tibetan includes 7 domains and 27 items. Confirmatory factor analysis suggests good fit for a unidimensional model for items within each domain but doesn’t support a unidimensional model for the entire instrument with all domains. Non-parametric and parametric item response theory analysis models show that for most items, the favorable response option (4 = definitely) is overwhelmingly endorsed, the discriminability parameter is over 1, and the difficulty parameters are all negative, suggesting that the items are most sensitive and specific for patients with poor primary care experience. Ongoing care is the strongest predictor of patient satisfaction. These findings suggest the need for some principles in adapting the tool to different health system contexts, more items measuring excellent primary care experience, and update of the four-point response options.

## Introduction

The contribution of primary care to health system performance has been widely examined nationally and internationally (Starfield *et al*., 2005; Kringos *et al*., 2013). A systematic review of 36 studies in low- and middle-income countries showed that strong primary care leads to improved and more equitable health outcomes, especially in infants and children (Macinko et al., 2009). Another study of 31 high-income countries in Europe showed that strong primary care system is associated with improved population health outcomes, reduced socioeconomic inequality in health outcomes, fewer unnecessary hospitalizations, and slower increases in the overall health-care expenditures (Kringos *et al*., 2013). Recognizing the value and effectiveness of primary care, many countries including China have identified primary care transformation as a major component of health reform.

Measuring progress towards high-performing primary health care can contribute to the achievement of sustainable development goals. Measuring the quality of primary care from patients’ perspective can provide actionable and comprehensive performance information to guide primary care reform efforts. Patients are the best evaluators of key aspects of their health care, including accessibility, continuity, interpersonal communication, respectfulness, family-centered care, whole-person care, and cultural sensitivity (Haggerty *et al*., 2007). To strengthen people-centered integrated primary care system building, there is increasing interests in patient experience measurement globally (Kruk *et al*., 2017). Many instruments have been developed, such as Primary Care Assessment Tool [(PCAT); Shi et al., 2001].

The PCAT developed by Barbara Starfield has been extensively used internationally (Shi *et al*., 2001). Inspired by the World Health Organization definition of primary care, the PCAT was originally developed to measure the extent to which primary care is achieved from user perspective in the United States. Seven primary care domains were included in the original English PCAT-adult version: first contact utilization and access, ongoing care, coordination with specialists, comprehensiveness of service available and provided, family centeredness, community orientation, and cultural competency (Shi *et al*., 2001).

The original English PCAT-adult version has been translated into many languages. The PCAT validation studies were mostly conducted in the following countries: China (Wang *et al*., 2014; Yang *et al*., 2013; Wang *et al*., 2015a; Wei *et al*., 2015; Mei *et al*., 2016), Canada (Haggerty *et al*., 2011b), South Korea (Lee *et al*., 2009), Argentina (Berra *et al*., 2013; Vazquez Pena *et al*., 2017), Spain (Pasarin *et al*., 2007; Berra *et al*., 2011), Brazil (Macinko *et al*., 2007), Japan (Aoki *et al*., 2016), Vietnam (Hoa et al., 2018), Turkey (Lağarlıa *et al*., 2014), and South Africa (Bresick et al., 2015). These validation studies suggest reasonable psychometric properties in the specific country context, but some common problems emerge, especially in factor structure and with response options. For example, factor analytic models do not support the underlying theoretical domains in several language versions (Lee *et al*., 2009; Yang *et al*., 2013; Mei *et al*., 2016). Response distributions tend to skew toward more favorable answers in most language versions, including English, compromising the formal statistical assumptions in the most psychometric analysis (Shi *et al*., 2001; Macinko *et al*., 2007; Pasarin *et al*., 2007; Lee *et al*., 2009; Berra *et al*., 2011; 2013; Haggerty *et al*., 2011a; 2011b; Yang *et al*., 2013; Lağarlıa *et al*., 2014; Wang *et al*., 2014; Bresick *et al*., 2015; Aoki *et al*., 2016; Mei *et al*., 2016; Vazquez Pena *et al*., 2017).

Some of these issues were also found in the Tibetan version of PCAT that was translated from one (Yang *et al*., 2013) of the three available Chinese versions (Yang *et al*., 2013; Wei *et al*., 2015; Wang *et al*., 2015a). The Chinese version developed by Yang *et al*. (2013) was adapted from adult version of PCAT longer version. The initial PCAT-Tibet included six domains of first contact, continuity, coordination, comprehensiveness, family-centeredness, and community orientation. The Tibet Autonomous Region is located in southwestern China, at an average elevation of 4000 m and an area about one eighth of China’s area, with a population of 3 million scattered over this large region. Since the national health reform initiated in China in 2009 (Yip *et al*., 2012) township health centers in Tibet were identified as the main primary care provider, and they received strong support from regional and national governments, including more health staff and salary increases, need-based training, and capital investments in infrastructure and necessary equipment. Actionable information about the effectiveness of these programs was needed for directing the resource allocation and to address the specific weakness in primary care service delivery.

The initial validation of PCAT-Tibetan suggested departures from the original factor structure and confirmed the skewed responses seen in other studies (Wang *et al*., 2014). For example, three original PCAT domains (first contact, continuity, and coordination) were split across five domains in the PCAT-Tibetan; and the original comprehensiveness domain was represented by two domains (Wang *et al*., 2014). These issues in psychometric properties in common with results from validation studies in other PCAT versions suggest a need for advanced psychometric analysis to examine the appropriateness of domains and items in relative to the original PCAT. In order to further examine the construct validity and reliability of PCAT-Tibetan version, this article reports on the results of confirmatory factor analysis to test the congruence between the theoretical primary care domains and the empirical results in Tibet, and on item response theory analysis to examine item performance and the appropriateness of item response options.

## Methods

This was a further analysis of the initial PCAT-Tibetan validation study that was conducted among 1386 patients who visited their primary care providers in three different types of health facilities in two prefectures in 2013. This survey was administered through face-to-face interview. The detailed information about sampling and data collection can be found in our previous publication (Wang *et al*., 2014).

Maximum likelihood (ML) imputation method was used to replace the missing values; match age, sex, education, self-rated health status; 295 respondents were excluded because of missing values for the matching variables, leaving 1091 respondents in subsequent analyses. Those excluded were more likely to be female and less educated. To examine the robustness of our conclusion, we excluded all respondents who had at least one missing value on any item (listwise deletion) and repeated all data analyses (*n* = 729), which did not alter any of the general conclusions. Values were also imputed for the ‘not sure/don’t remember’ response option as an alternative to attributing the pre-set value of 2.5. In general, estimates produced with listwise deletion are less efficient than other methods of handling missing data. Therefore, we reported only results using database with ML imputation (Enders, 2001).

In user-evaluation research, it is common to treat report and rating values as quasi-cardinal (DeVellis, 1991). The items measuring patient experience in this study were strictly ordinal level, so we treated them as interval level, which was consistent with previous validation studies of original PCAT version in the United States (Shi *et al*., 2001) and the PCAT versions in other languages (Lee *et al*., 2009; Haggerty *et al*., 2011b; Yang *et al*., 2013; Aoki *et al*., 2016; Mei *et al*., 2016). This approach was also used by the validation study of World Health Organization’s health system responsiveness survey (Valentine *et al*., 2007). Therefore, the four-point Likert response scale in PCAT-Tibetan version was treated as continuous variable.

First, the inter-item correlation and exploratory factor analysis (principle component analysis) by domain was conducted to flag items with low correlations (Pearson <0.20) or low factor loading (<0.30) for potential deletion. We repeated the exploratory factor analysis after deleting each item with low factor loading until all retained items had a factor loading of at least 0.30. The results of exploratory factor analysis guided subsequent confirmatory factor analysis.

Then confirmatory factor analysis using structural equation modeling was used to test the goodness of fit of the items to the theoretical domains of the original PCAT. Subsequently, confirmatory factor models were adjusted iteratively based on fit and judgment until the goodness-of-fit statistics were optimized. We also assessed the entire instrument including all domains in our data analysis. First, we included all domains in confirmatory factor analysis. Then we only included the four core domains (first contact, continuity, coordination, and comprehensiveness) in confirmatory factor analysis. The following goodness-of-fit statistics were used: normed fit index (NFI) ≥0.9 indicating good fit, comparative fit index (CFI) ≥0.9 indicating good fit, standardized root mean square residual (SRMR) ≤0.05 indicating acceptable fit.

For each domain confirmed as being unidimensional, we examined the distribution of response options of individual items within each domain based on domain performance using nonparametric item response theory analysis. However, we cannot get the exact information of each item performance. To be more precise, two parameter estimates (discriminability and difficulty) were subsequently generated using Samejima’s (1969) grade response model.

We estimated the correlations between domains to examine how domains were related and whether they demonstrated distinctiveness.

Finally, we used logistic regression modeling to examine how primary care domains were associated with patients’ satisfaction with service attitude (one item) and perceived technical quality (one item) of their primary care provider. The five-point Likert response scale for the two items was dichotomized to indicate satisfaction (very satisfied or satisfied) versus dissatisfaction. All domains were put in the same model, and age, sex, education, and self-rated health status were included in the model as covariates. Education level was categorized into three groups: illiterate, primary school, junior high school and above. Self-rated health status was measured by a visual analogue scale with end points of 0 and 100, where 0 corresponds to ‘the worst health status’, and 100 corresponds to ‘the best health’.

Descriptive, correlation, and exploratory factor analysis were conducted with SPSS 22.0. ML imputation and confirmatory factor analysis were conducted with LISREL9.1. Nonparametric and parametric item response theory analyses was conducted with SPSS 22.0 and MULTILOG 7.03.

## Results

Table 1 summarizes the response distribution and descriptive statistics of each item in initial PCAT-Tibetan. The percentage of true missing values for each item ranged from 0.9% to 4.4%. Most items are slightly negatively skewed, and with over 50% respondents reporting the most favorable response option ‘4 = Definitely’ on 22 of 36 items. The percentage of respondents choosing the response option of ‘not sure/don’t remember’ is higher, particularly for items in first contact access, coordination, and community orientation, suggesting that patients may not be the best information source for these domains or may not have direct experience with all aspects elicited.

**Table 1.** Statements and descriptive statistics by items in PCAT-Tibetan version^a^

Domains	Items/statements ‘How likely is it that…?’	True missing % (n)	Response options % (*n*)					Mean	SD		
			1 = Definitely not	2 = Probably not	3 = Probably	4 = Definitely	NS/DNK			Median	Skewness
**First contact Utilization**	**FCU1.** See your provider first for a regular general checkup	1.5% (21)	2.8% (39)	5.0% (69)	16.8% (233)	70.6% (978)	3.3% (46)	3.64	0.70	4.00	−2.07
	**FCU2.** See your provider first for a new health problem	1.4 % (19)	1.2% (16)	3.6% (50)	22.7% (314)	67.0% (929)	4.2% (58)	3.64	0.62	4.00	−1.88
**First contact access**	**FCA1.** Being seen same day when provider is open	0.9 % (13)	1.2 % (16)	3.3% (46)	17.5 % (243)	74.2 % (1028)	2.9% (40)	3.73	0.57	4.00	−2.32
	**FCA2.** Get advice over phone when provider is open	1.7% (24)	11.1 % (154)	13.4% (186)	29.4% (407)	39.2% (544)	5.1% (71)	3.04	1.03	3.00	−0.75
	**FCA3.** Get advice over phone when provider is closed	2.2% (30)	17.6% (244)	13.3% (185)	21.4 % (296)	34.3% (476)	11.2% (155)	2.83	1.17	3.00	−0.46
	**FCA4.** Being seen same day when provider is closed	2.1% (29)	2.5% (35)	7.6% (105)	14.4 % (199)	66.7% (924)	6.8% (94)	3.61	0.74	4.00	−1.95
	**FCA5.** Wait over 30 minutes before being checked	2.3% (32)	29.1% (404)	14.9% (207)	24.2% (336)	23.4% (324)	6.0% (83)	2.56	1.18	2.00	0.02
	**FCA6.** Difficulty to get needed medical care	2.2% (31)	43.9 % (609)	12.0 % (166)	16.5 % (228)	19.3% (267)	6.1% (85)	2.88	1.23	3.00	−0.47
**Ongoing care**	**OC1.** Being taken care by the same doctor or nurse	1.7% (24)	13.7% (190)	17.6 % (244)	25.5% (354)	34.7% (481)	6.7% (93)	2.90	1.09	3.00	−0.52
	**OC2.** Being explained with patience and in ways that you understand	2.5% (35)	1.7% (24)	2.3 % (32)	17.4 % (241)	73.0% (1012)	3.0% (42)	3.74	0.56	4.00	−2.60
	**OC3.** Talk to the doctor or nurse who knows you best	2.7% (37)	4.3% (59)	7.4% (102)	32.3% (448)	50.9% (705)	2.5% (35)	3.42	0.79	4.00	−1.36
	**OC4.** Being given enough time to talk	1.9% (26)	2.0% (28)	2.0% (28)	17.4% (241)	74.5% (1032)	2.2% (31)	3.72	0.60	4.00	−2.63
	**OC5.** Your provider know if you had trouble paying for medicines	1.4% (19)	6.5% (90)	16.5% (229)	24.3% (337)	46.6% (646)	4.7% (65)	3.18	0.96	3.00	−0.84
	**OC6.** Your provider know all the treatment and medications you are taking	2.2% (31)	2.7% (38)	2.7 % (37)	20.9% (290)	68.8% (954)	2.6% (36)	3.67	0.64	4.00	−2.25
	**OC7.** Change your provider if needed	1.6% (22)	8.7% (120)	7.6% (105)	37.1% (514)	40.8% (565)	4.3% (60)	1.81	0.92	2.00	1.07
	**OC8.** Being followed up by your provider	2.4% (33)	5.1 % (70)	10.5 % (146)	25.3% (350)	55.7% (772)	1.1% (15)	3.39	0.83	4.00	−1.17
**Coordination**	**CD1.** Discuss with you different places you could go	1.8% (25)	3.0% (42)	2.3% (32)	20.7% (287)	68.8% (953)	3.4% (47)	3.66	0.67	4.00	−2.33
	**CD2.** Write down information for your referral	2.0% (28)	3.8% (53)	3.8% (53)	29.5% (409)	56.9% (788)	4.0% (55)	3.50	0.75	4.00	−1.66
	**CD3. T**alk with you about what happened after referral	2.5% (35)	4.9% (68)	6.2% (86)	21.6% (299)	58.6% (812)	6.2 % (86)	3.49	0.83	4.00	−1.68
	**CD4.** Be interested in the quality of care after referral	2.2% (30)	6.1% (85)	5.1% (70)	25.7% (356)	53.5% (741)	7.5% (104)	3.42	0.86	4.00	−1.55
**Comprehensiveness**	**CP1.** Advice about healthy foods and unhealthy foods or getting enough sleep	1.9% (27)	0.6% (8)	5.4% (75)	20.6 % (285)	70.8% (981)	0.7% (10)	3.68	0.60	4.00	−1.89
	**CP2.** Home safety, like getting air to the room regularly and storing medicines safely	2.4% (33)	7.5% (104)	6.0% (83)	26.3% (364)	55.7% (772)	2.2% (30)	3.39	0.90	4.00	−1.47
	**CP3.** Ways to handle pressures and work and interpersonal conflicts	2.2% (31)	3.7% (51)	7.4% (102)	34.7% (481)	48.7% (675)	3.3% (46)	3.41	0.77	4.00	−1.31
	**CP4.** Ways to handle family conflicts that may arise from time to time	2.4% (33)	4.5% (63)	9.0% (125)	29.4% (408)	51.7% (716)	3.0% (41)	3.41	0.83	4.00	−1.37
	**CP5.** Advice about appropriate exercise for you	2.4% (33)	4.2% (58)	4.8% (67)	21.9 % (304)	65.7% (911)	0.9% (13)	3.58	0.76	4.00	−1.98
	**CP6.** Test for cholesterol levels in your blood	2.5% (34)	10.1% (140)	20.9% (289)	22.2% (308)	41.9% (581)	2.5% (34)	3.03	1.05	3.00	−0.62
	**CP7.** Test for blood pressure	2.4% (33)	5.1% (70)	10.5% (146)	25.3 % (350)	55.7% (772)	1.1% (15)	3.39	0.86	4.00	−1.31
	**CP8.** Care for women’s health or men’s health and do regular check-up	3.1% (43)	8.9% (124)	12.5% (173)	22.4% (310)	44.7% (620)	8.4% (116)	3.20	0.99	4.00	−0.98
**Family centeredness**	**FC1.** Ask about family members’ opinions when planning treatment	2.9% (40)	2.5% (35)	7.0 % (97)	32.8% (454)	51.8% (718)	3.0% (42)	3.45	0.73	4.00	−1.31
	**FC2.** Introduce types of medicines before prescription	2.7% (37)	2.3% (32)	6.3% (88)	28.6% (397)	58.9% (817)	1.1% (15)	3.53	0.70	4.00	−1.51
	**FC3.** Ask about illnesses that run in your family	3.0% (42)	4.3% (59)	4.6 % (64)	14.9% (207)	70.8% (981)	2.4% (33)	3.63	0.75	4.00	−2.19
	**FC4.** Meet with your family members if you thought it would be helpful	3.3% (46)	6.2% (86)	6.6% (91)	29.0% (402)	51.3% (711)	3.6% (50)	3.36	0.89	4.00	−1.37
**Community orientation**	**CO1.** Make home visits	2.5% (34)	10.2% (142)	21.9% (303)	27.7% (384)	33.7% (467)	4.0% (56)	2.93	1.02	3.00	−0.48
	**CO2.** Know the important health problems in your community	3.3% (46)	11.3% (157)	10.2% (141)	24.5% (340)	41.6% (577)	9.0% (125)	3.13	1.05	3.00	−0.93
	**CO3.** Surveys of patients to see if the services are meeting people’s needs	4.4% (61)	9.2% (127)	11.9% (165)	20.6% (285)	45.9% (636)	8.1% (112)	3.20	1.01	4.00	−0.97
	**CO4.** Survey in the community to find out about health problems	2.7% (38)	9.1% (126)	12.2% (169)	25.7% (356)	41.8 % (580)	8.4% (117)	3.14	1.00	3.00	−0.87

a *n* = 1386. Mean, median, and standard deviation were calculated using the database with missing values imputed by maximum likelihood method (*n* = 1091).·Item labels being struck through (eg, FCA5) are deleted from the final version.

NS/DNK = not sure/don’t know.

For first contact access, two of the six items were deleted because of the unacceptable goodness-of-fit statistics (FCA5 and FCA6); and two items fit better in first contact utilization (FCA1 and FCA4), leaving only two items in first contact access and four in first contact utilization. In the eight-item ongoing care, two items were deleted because of unacceptable goodness-of-fit statistics (OC1 and OC7); and a further item (OC8) was removed to improve the goodness-of-fit statistics, leaving a five-item ongoing care scale. The eight-item comprehensiveness subscale showed unacceptable goodness-of-fit statistics on a single factor (NFI = 0.27, CFI = 0.27, SMRM = 0.19); and after removing four of the poorest fitting items, a final four-item comprehensiveness subscale demonstrated good fit (NFI = 0.98, CFI = 0.99, SMRM = 0.02). There is no change in items in other domains. The detailed iterative process to finalize the original factor structure was reported in Supplemental Table S1.

Table 2 shows the goodness-of-fit statistics of confirmatory factor analysis for each domain in the final structural model. The fit statistics of first contact utilization and coordination do demonstrate a moderate fit. For ongoing care, comprehensiveness, family centeredness, and community orientation, all the four confirmatory factor analysis models demonstrate a good fit. The empirical results suggest a best final structure for the PCAT-Tibetan of 7 domains and 27 items instead of 36. At least three items are needed for confirmatory factor analysis. First contact access only includes two items, so we did not have confirmatory factor analysis results.

**Table 2.** Summary of results from final model in confirmatory factor analysis for each domain and internal consistency^a^ ^,^ ^b^

Separate domain models	Initial number of items	Final number of items	Factor loading	Chi square	DF	SRMR^c^	NFI^d^	CFI^e^	Domain score, mean	Domain score, SD	Domain score, median	Cronbach *α*
First contact utilization	2	4	0.62–0.90	160.40	2	0.057	0.848	0.849	3.66	0.48	4.00	0.70
First contact access	6	2	‐	‐	‐	‐	‐	‐	2.94	0.95	3.00	0.66
Ongoing care	8	5	0.50–0.79	31.44	5	0.025	0.950	0.957	3.55	0.47	3.60	0.66
Coordination	4	4	0.76–0.88	266.90	2	0.070	0.833	0.833	3.52	0.61	3.75	0.80
Comprehensiveness	8	4	0.62–0.87	20.21	2	0.019	0.984	0.986	3.27	0.73	3.50	0.77
Family centeredness	4	4	0.66–0.82	6.83	2	0.013	0.988	0.992	3.49	0.56	3.75	0.70
Community orientation	4	4	0.80–0.89	126.02	2	0.040	0.943	0.943	3.10	0.85	3.25	0.85

a *n* = 1091. At least three items are needed for confirmatory factor analysis: first contact access only includes two items, so we did not have confirmatory factor analysis results.

b Weighted least square estimation method was used for confirmatory factor analysis using LISREL version 9.1.

c SRMR = standardized root mean square residual; ≤0.08 indicating acceptable fit.

d NFI = normed fit index; ≥0.9 indicating good fit.

e CFI = comparative fit index; ≥0.9 indicating good fit.

However, either the model including all domains (NFI = 0.46, CFI = 0.46, SMRM = 0.23) or the model including only the four core domains (NFI = 0.55, CFI = 0.56, SMRM = 0.14) shows unacceptable goodness-of-fit statistics on a single factor, which suggest that the domains included in original PCAT may not measure a common single construct in Tibet context, and it is not appropriate to report a total score.

The mean of each domain score is lower than the median and negatively skewed, indicating most of patients reporting favorable response answers. The first contact utilization score is the highest (3.66±0.48), while the first contact access score is the lowest (2.94±0.95). Cronbach *α* is over 0.70, indicating good internal consistency of items for all domains except first contact access (0.66) and ongoing care (0.66).

**Figure 1.**
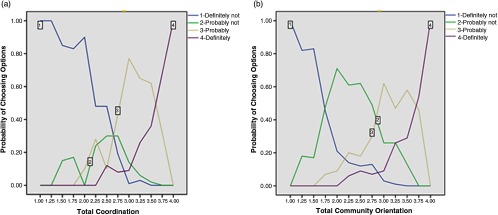
Response graph by non-parametric item response theory analysis contrasting poorly and well-performing items. (a) Option characteristic curves for item CD4 in coordination ‘Was your primary care provider interested in the quality of care there?’ are modeled as a function of total scores on these measures (bottom axis). Results show difficulties with some options from this item. The probability of endorsing option 2 is relatively small compared to other options. (b) Option characteristic curves for item C01 in community orientation ‘Does your primary care provider do survey in the community to find out about health problems he or she should know about?’ are modeled as a function of total scores on these measures (bottom axis). Results show that this item performs well relative to other items.

Non-parametric item response theory graphs were modeled on each unidimensional domain to provide further insight into item performance and reliability. In most items, the option characteristic curve for the response option ‘2 = Probably not’ is overshadowed by other options (see example in Figure 1a), indicating that nowhere along the primary care experience continuum was this option more likely to be chosen than other options, raising the question of the appropriateness of a four-point response scale. Only a few items perform optimally, such that the probability of choosing each response option is highest in a unique zone of primary care experience continuum, reflecting clearly ordinal response behavior appropriate to the assigned value for each option. Figure 1b shows a well-performing item from community orientation. A problem common to all items is that the extreme response option ‘4 = definitely’ covers a large area of primary care experience continuum and is most likely to be endorsed, even at below-average primary care experience level, suggesting that additional response options may be desirable.

The results from parametric item response theory analysis provide further evidence to confirm the findings from non-parametric item response theory analysis (Table 3). The discriminability parameter is over 1 for all items except one item on ongoing care, indicating that response options discriminate well between low and high levels of primary care experience. However, the difficulty parameters for almost all items are negative, indicating that positive ratings (b2, b3) are endorsed at less than average performance, reinforcing the pattern observed in Figure 1a. Likewise the information curves show that the majority of items are most informative in the negative zone of the underlying construct. Only CO1, illustrated in Figure 1b, shows that each response option corresponds to a distinct zone of the domain, including the most positive experience. Together these suggest that the items are most sensitive and specific for patients with poor primary care experience.

**Table 3.** Item performance for each item within its domain, showing discriminability (a) and difficulty (b) parameters^a^

Domain	Item	Item-scale correlation	a^b^	b1^b^	b2^b^	b3^b^
**First contact utilization**	FCU1	0.49	2.17	−2.58	−1.85	−0.89
	FCU2	0.58	4.08	−2.36	−1.76	−0.62
	FCA1	0.48	1.97	−3.19	−2.29	−1.07
	FCA4	0.41	1.21	−3.47	−2.24	−1.11
**First contact access**	FCA2	0.50	‐	‐	‐	‐
	FCA3	0.50	‐	‐	‐	‐
**Ongoing care**	OC2	0.47	2.41	−2.67	−2.31	−1.00
	OC3	0.46	1.56	−2.68	−1.84	−0.25
	OC4	0.43	2.07	−2.67	−2.35	−1.03
	OC5	0.35	0.99	−3.04	−1.38	−0.04
	OC6	0.45	1.91	−2.75	−2.24	−0.89
**Coordination**	CD1	0.52	1.69	−2.70	−2.38	−0.89
	CD2	0.58	1.92	−2.38	−1.86	−0.39
	CD3	0.67	3.69	−1.77	−1.31	−0.47
	CD4	0.66	3.19	−1.69	−1.33	−0.31
**Comprehensiveness**	CP2	0.46	1.35	−2.36	−1.74	−0.42
	CP6	0.67	2.96	−1.42	−0.56	0.11
	CP7	0.60	2.57	−1.97	−1.19	−0.30
	CP8	0.56	1.89	−1.82	−1.01	−0.07
**Family centeredness**	FC1	0.47	1.79	−2.79	−1.82	−0.26
	FC2	0.55	2.60	−2.47	−1.70	−0.42
	FC3	0.46	1.91	−2.38	−1.81	−0.96
	FC4	0.47	1.57	−2.21	−1.61	−0.27
**Community orientation**	CO1	0.67	2.47	−1.52	−0.48	0.36
	CO2	0.72	3.15	−1.29	−0.80	0.03
	CO3	0.65	2.29	−1.63	−0.85	−0.08
	CO4	0.72	2.93	−1.52	−0.76	0.06

a *n* = 1091. Domains were scored by averaging the value of individual items. Item response scale ranged from 1 to 4. Higher score meant better patient experience.

b IRT parameter estimates were generated using Samejima’s (1969) grade response model and using IRT software MULTILOG 7.03.

a = discriminability; value >1 indicating minimal discriminability. b = difficulty; highest probability of endorsing.

The Pearson correlations between the domains indicate the distinctiveness of each domain. Correlation coefficients between domains range from 0.23 to 0.61 and are lower than Cronbach *α* of each domain. Coordination is most highly correlated with family centeredness (0.61) and ongoing care (0.60). First contact utilization is also highly correlated with family centeredness (0.54) and ongoing care (0.55). First contact access, comprehensiveness, and community orientation have lower correlation with other domains.

**Table 4.** Odds ratios (95% confidence intervals) of patient satisfaction associated with each unit increase in primary care domain score after adjusting for sex, age, education, and health status in logistic model

Domains	‘Are you satisfied with the service attitude of health staff in your primary care provider?’ *n* = 1048			‘Are you satisfied with the technical quality in your primary care provider?’ *n* = 1051		
	Odds ratios	95% confidence intervals	*P*-value	Odds ratios	95% confidence intervals	*P*-value
First contact utilization	2.94	(1.92, 4.50)	<0.001	0.88	(0.57, 1.35)	0.555
First contact access	1.26	(0.99, 1.61)	0.058	1.65	(1.33, 2.05)	<0.001
Ongoing care	2.65	(1.61, 4.38)	<0.001	3.93	(2.44, 6.31)	<0.001
Coordination	1.10	(0.75, 1.63)	0.619	1.77	(1.25, 2.51)	0.001
Comprehensiveness	1.16	(0.85, 1.58)	0.352	1.17	(0.89, 1.54)	0.272
Family centeredness	1.30	(0.83, 2.04)	0.257	0.84	(0.55, 1.28)	0.407
Community orientation	1.05	(0.78, 1.40)	0.763	1.03	(0.80, 1.32)	0.850

Finally, Table 4) shows the extent to which a unit increase in each domain score increases the odds of patient satisfaction. Although most patients are satisfied with the service attitude (82.6%) and the technical quality (80.2%) of their primary care provider, a higher PCAT score generally is associated with higher satisfaction. For example, every unit increase in ongoing care score increases the likelihood of being satisfied with service attitude by 2.65 times.

## Discussion

This advanced psychometric analysis of the PCAT-Tibetan versions provides further insight into some of the problematic psychometric properties found in the initial validation analysis, and it suggests avenues to improve the metric performance of the tool. Despite the metric problems, the PCAT-Tibetan domains of first-contact, ongoing care, and coordination with specialists are associated with an increased likelihood of patient satisfaction. This illustrates the potential of the PCAT and underlines the importance of improving the tool to address some of the metric problems. Some of the problems, such as the skewness of item response distribution found in many items, are shared with the original PCAT and other versions; and our results suggest some solutions that could improve performance. Others may be specific to the PCAT-Tibetan version – such as the non-optimal resolution of items relating to First Contact constructs – suggest the need for some principles in adapting the tool to different health system contexts.

The widespread use of the PCAT to evaluate primary care in many countries provides an opportunity to compare primary care across different contexts and to support a worldwide movement to improve primary care. Our results show not only the association of the PCAT-Tibetan with patient satisfaction but also other analysis that demonstrated the capacity to distinguish between health-care organizations in Tibet and other regions in China (Wang *et al*., 2013; McCollum *et al*., 2014; Wang *et al*., 2015b; Hu *et al*., 2016; Feng *et al*., 2017). Other studies have shown the capacity of different versions of the PCAT to differentiate between delivery models. For instance, in the United States, community health centers have been showed to provide better quality primary care than health maintenance organizations, especially in continuity, coordination, and comprehensiveness (Shi *et al*., 2003). Patients mainly receiving care from private general practitioners in Hong Kong reported better primary care experiences than those mainly receiving care from public general outpatient clinics, especially in accessibility and interpersonal relationships (Wong *et al*., 2010). In South Korea, among four types of primary care clinics staffed by family physicians, health cooperative clinics displayed the best primary care performance, while public health center clinics showed the worst performance (Sung *et al*., 2010).

The negative skewness of item response distribution in many items was noted in the original validation of the long PCAT version (Shi *et al*., 2001) and has been found in most other validation studies (Macinko *et al*., 2007; Lee *et al*., 2009; Berra *et al*., 2011; Haggerty *et al*., 2011b; Berra *et al*., 2013; Yang *et al*., 2013; Lağarlıa *et al*., 2014; Aoki *et al*., 2016; Mei *et al*., 2016) but may be more extreme in contexts such as China where low literacy requires face-to-face administration (Haggerty *et al*., 2011a). The item response theory analysis shows that the PCAT is most reliable in identifying negative experience of care, but the low information yield in the above average range of the domains means that it will have limited sensitivity for detecting improvements in care. Some researchers suggested that new response categories should be developed to minimize the favored response (Yang *et al*., 2013), but this would be challenging using the current response scale as it is difficult to imagine an intermediate category between ‘probably yes’ and ‘definitely yes’. The acceptable discriminability parameters for most items reflect adequate capacity to discriminate between poor and average performance on domains even though the four-point response values are not optimal. This suggests that some form of dichotomous scoring could be applied to each item to give greater weight to the more informative negative responses rather than averaging across all response options. This approach is used in a Brazil study (Macinko *et al*., 2007) and also in the Consumer Assessment of Healthcare Providers and Systems (CAHPS) (Dyer *et al*., 2012). Finally, to increase discriminability and improve the potential for sensitivity to improvement, it would be good to develop more items to measure excellent primary care experience. The community orientation item about home visits (CO1) is an example of an item that discriminates clearly between average and good primary care.

Another metric issue results from offering the ‘not sure/don’t remember’ option. The high rate of endorsing this response option is common in many language versions, especially in Asian countries including Korean, Japanese and Chinese. (Lee *et al*., 2009; Yang *et al*., 2013; Wang *et al*., 2014; Aoki *et al*., 2016; Mei *et al*., 2016). Although respondents appreciate having such response option (Haggerty *et al*., 2011a), its management is analytically challenging. Although the PCAT scoring manual suggests attributing a value of 2.5, this is not supported for all items in a previous response theory analysis of English and French versions of the PCAT (Haggerty *et al*., 2011b). A more usual practice would be to treat the values as missing and attribute values using more ML methods. However, the approach used (excluding these as missing values) will impact on the factor analysis and the subsequent conclusions about the validity of PCAT version. Again, this seems to call for further work and international collaborations on response options that accord with patient experience in different contexts.

Collaborative international work could also address principals for measuring and/or comparing domains that affected health system specificities, for instance, in the access domain. First contact in the PCAT-Tibetan fails to meet optimal psychometric standards of construct validity and internal consistency. Similar problems were also found in other PCAT validation studies in China (Yang *et al*., 2013; Mei *et al*., 2016) and other countries in Asia (Lee *et al*., 2009; Lağarlıa *et al*., 2014; Aoki *et al*., 2016). Two studies of the PCAT-Chinese version found that Cronbach *α* was only 0.38 (Mei *et al*., 2016) and 0.48 (Yang *et al*., 2013) for first contact utilization; similar values were found in the PCAT-Turkish (Lağarlıa *et al*., 2014). The validation of PCAT-Korean concluded that first contact could not be assessed using a traditional scale with multiple correlated items, and it was treated to be a composite domain consisting of five independent single-item subscales (Lee *et al*., 2009). The exploratory factor analysis of PCAT-Japanese collapsed first contact utilization and first contact access into one scale (Aoki *et al*., 2016). These results are not surprising, given that access reflects the fit between how services are organized and the perceived need of the population (Penchansky and Thomas, 1981; Khan and Bhardwaj, 1994). Consequently, the access dimension will be sensitive to contextual differences in how services are organized. Although these subscales perform well in studies in the North American context (Shi *et al*., 2001; Haggerty *et al*., 2011c), the first contact items in original PCAT may not be appropriate in other countries with different health system organization and patient expectations. Making appointment in advance and gatekeeping by primary care worker do not pertain to many countries. For example, geographical accessibility is a major constraint for local people to get health-care services in Tibet, but it was not addressed in the PCAT. Context-specific items should be explored based on the operational definitions of first contact. Similar issues are likely to pertain to comprehensiveness and coordination with specialists.

In contrast, the domain of ongoing care most strongly predicts patient satisfaction with primary care provider in Tibet. This is consistent with a previous systematic review showing that the most important determinants of satisfaction are the interpersonal relationships and their related aspects of care (Crow *et al*., 2002). Recognizing the benefit of continuity of care, China is developing a family doctor contract service model to build a long-term trust doctor–patient relationship. Under this model, residents can sign a contract with a family doctor working at a community health center and be eligible for a service contract package including basic medical care, public health, and health management service. Although countries differ in the organizational support of continuing provider–patient relationships, our study and studies in other countries point to the value of a stable long-term relationship and mutual interactive communication between patient and primary care workers (Baker *et al*., 2003; Paddison *et al*., 2015). Nonetheless, measures may be improved by accounting for cultural specificities in interpersonal communication and therapeutic relationships that impact on both patient experience and outcomes.

This study contributes to the growing international work supporting the relevance and need for valid and reliable measures of the patient experience of health care. Several lessons from PCAT-Tibetan validation study may be shared with our colleagues in low- and middle-income countries. First, the items or content of instruments from developed countries may not be appropriate in other countries. Some specific features of local primary care system may not be reflected in the translated instruments. For example, no items in PCAT could reflect the feature of geographical accessibility, which is a major aspect in Tibet. Some items in comprehensiveness domain are not appropriate in Tibet. Therefore, instead of adapting existed instrument directly, qualitative research is needed to understand the local population preference of primary care first. Second, more items to measure excellent primary care experience should be developed. Most existing items have adequate capacity to discriminate between poor and average performance on different primary care domains. However, items that discriminate clearly between average and good primary care are needed. Further research is required to explore the characteristics of some exemplars with good primary care experience and what the good primary care is from their narratives. Third, the choice of response categories should be careful. The current four-point response scale and its wording in PCAT may not be appropriate in some countries. Several factors could be considered when exploring the appropriate response categories, such as literacy level, response tendency, and judgment making of local population. This could be done through qualitative research.

Finally, a summary score of overall primary care experience including all domains, which is often the most used metric when assessing a health system, is not supported by our analysis of PCAT-Tibetan version. However, this psychometrically validated 27-item Tibetan version of PCAT will be useful in monitoring and evaluating the performance of primary care system in Tibet in specific areas, especially in accessibility, continuity, and coordination, which are the priorities of current health reform efforts in Tibet. The health service research is underdeveloped in Tibet, and there is no instrument measuring patient experience that could be used when this study was conducted. We hope this study could bring more researchers’ attention into primary care performance evaluation in Tibet. We also recognize that different policy interventions to achieve primary care functions are inter-related, but each policy has its own priorities. For example, the family doctor contract service model is being developed and expanded now to improve performance in accessibility and continuity; and the transformation of tiered health service delivery system aims to promote collaboration between different health-care providers and to improve coordination. Under this context, PCAT-Tibetan version is a potential useful instrument to evaluate the effectiveness of these policy interventions.

## Supplementary material

For supplementary material accompanying this paper visit https://doi.org/10.1017/S1463423619000239. click here to view supplementary material
