# Supplementary material for: Development of primary care assessment tool–adult version in Tibet: implication for low- and middle-income countries
Source: Prim Health Care Res Dev. 2019 Jul 1;20:e94. doi: 10.1017/S1463423619000239 (PMC6609993; doi:10.1017/S1463423619000239)
Supplement: Supplementary file 1 [file S1463423619000239sup001.docx]

**Supplemental Table 1 Iterative process to finalize the original factor structure based on the results of confirmatory factor analysis (CFA) (n=1091)**

|  |  | **Inter-item correlation ^a^** | **Factor loading ^b^** | **Interactive process to identify the optimal factor structure ^c^** |
| --- | --- | --- | --- | --- |
| **Domains** | **Items** |  |  |  |
| **First Contact Utilization (FCU)** | FCU1 | 0.56 | 0.731 | 1. All six items in FCA were included in one confirmatory factor analysis model first. However, CFA model showed unacceptable goodness of fit statistics. NFI=0.17, CFI=0.17, SMRM=0.20.  2. FCA 5 and FCA 6 were flagged for potential deletion because of lower factor loading by exploratory factor analysis. Therefore, FCA including FCA1, FCA2, FAC3, and FCA4 was examined again using confirmatory factor  analysis, but the results still showed unacceptable goodness of fit statistics. NFI=0.64, CFI=0.64, SMRM=0.10.  3. Considering higher inter-item correlation among the six items (0.15-0.56): FCU1 and FCU2 in FCU, FCA1, FCA2, FAC3, and FCA4 in FCA, these six items were analyzed using exploratory factor analysis model. The results showed two factors: Factor 1 including FCU1, FCU2, FCA1, FCA4; Factor 2 including FCA2, FAC3.  4. Two confirmatory factor analysis models were established to examine if the two factors measure one  common latent variable. One model was with correlated factors, and the other model with second-order  latent variable. Both of CFA models showed unacceptable goodness of fit statistics. NFI=0.57, CFI=0.57,  SMRM=0.13.  5. Based on the above analysis results, two factors were identified: FCU including FCU1, FCU2, FCA1, FCA4; and FCA including FCA2, FAC3. |
|  | FCU2 | 0.56 | 0.731 |  |
| **First Contact Access (FCA)** | FCA1 | 0.07-0.25 | 0.366 |  |
|  | FCA2 | 0.12-0.51 | 0.727 |  |
|  | FCA3 | 0.00-0.51 | 0.550 |  |
|  | FCA4 | 0.11-0.39 | 0.412 |  |
|  | FCA5 | 0.03-0.45 | 0.208 |  |
|  | FCA6 | 0.00-0.45 | 0.234 |  |
| **Ongoing Care (OC)** | OC1 | 0.02-0.27 | 0.233 | 1. All eight items in OC were included in one confirmatory factor analysis model first. However, CFA model showed unacceptable goodness of fit statistics. NFI=0.68, CFI=0.70, SMRM=0.07.  2. OC1 and OC7 in OC were flagged for potential deletion because of lower correlation and lower factor loading by exploratory factor analysis. Therefore, OC without OC1 and OC7 was examined using confirmatory factor analysis, but the results showed only moderate goodness of fit statistics: NFI=0.86, CFI=0.87, SMRM=0.04.  Specifically, OC5 and OC8 were flagged for potential deletion because of low factor loading in the above confirmatory factor analysis model.  3. Ongoing Care without OC1, OC5 and OC7 was examined using confirmatory factor analysis, but the results  still showed only moderate goodness of fit statistics. NFI=0.88, CFI=0.88, SMRM=0.04.  4. Ongoing Care without OC1, OC7 and OC8 was examined using confirmatory factor analysis, the goodness  of fit statistics gained significant improvement- Chi-Square value decreased from 105.50 to 31.44. NFI=0.95,  CFI=0.96, SMRM=0.02. Therefore, OC5 was retained and OC8 was removed.  5. OC included five items: OC2, OC3, OC4, OC5, and OC6. |
|  | OC2 | 0.06-0.43 | 0.577 |  |
|  | OC3 | 0.11-0.37 | 0.618 |  |
|  | OC4 | 0.02-0.43 | 0.509 |  |
|  | OC5 | 0.19-0.29 | 0.468 |  |
|  | OC6 | 0.10-0.41 | 0.583 |  |
|  | OC7 | 0.11-0.19 | -0.188 |  |
|  | OC8 | 0.11-0.39 | 0.559 |  |
| **Coordination** | CD1 | 0.38-0.48 | 0.582 | Exploratory factor analysis suggested one factor was optimal.  Confirmatory factor analysis model showed moderate goodness of fit statistics: NFI=0.84, CFI=0.84, SMRM=0.07. |
|  | CD2 | 0.44-0.49 | 0.660 |  |
|  | CD3 | 0.40-0.63 | 0.785 |  |
|  | CD4 | 0.38-0.63 | 0.774 |  |
| **Comprehensiveness** | CP1 | 0.25-0.44 | 0.459 | 1. All eight items were included in one confirmatory factor analysis model, and the results showed  unacceptable goodness of fit statistics. NFI=0.27, CFI=0.27, SMRM=0.19.  2. Considering the low correlation of CP3 and CP4 with other items, confirmatory factor analysis was  repeated after removing CP3 and CP4. The goodness of fit statistics were still unacceptable: NFI=0.78,  CFI=0.78, SMRM=0.09. CP1 and CP5 were flagged for potential deletion because of low factor loading in the  above confirmatory factor analysis model.  3. Comprehensiveness including 4 items (CSP2 CSP6 CSP7 CSP8) was examined using confirmatory factor  analysis, the goodness of fit statistics gained significant improvement- Chi-Square value decreased from  2008.90 to 21.10. NFI=0.98, CFI=0.99, SMRM=0.02.  4. Comprehensiveness domain included four items: CP2 CP6 CP7 CP8. |
|  | CP2 | 0.17-0.40 | 0.498 |  |
|  | CP3 | 0.20-0.51 | 0.469 |  |
|  | CP4 | 0.17-0.51 | 0.481 |  |
|  | CP5 | 0.20-0.44 | 0.523 |  |
|  | CP6 | 0.20-0.58 | 0.692 |  |
|  | CP7 | 0.23-0.58 | 0.679 |  |
|  | CP8 | 0.27-0.51 | 0.654 |  |
| **Family Centeredness (FC)** | FC1 | 0.34-0.45 | 0.596 | Exploratory factor analysis suggested one factor was optimal.  Confirmatory factor analysis model showed acceptable goodness of fit statistics: NFI=0.99, CFI=0.99, SMRM=0.01. |
|  | FC2 | 0.41-0.45 | 0.716 |  |
|  | FC3 | 0.33-0.42 | 0.564 |  |
|  | FC4 | 0.33-0.41 | 0.571 |  |
| **Community Orientation (CO)** | CO1 | 0.48-0.63 | 0.743 | Exploratory factor analysis suggested one factor was optimal.  Confirmatory factor analysis model showed acceptable goodness of fit statistics: NFI=0.94, CFI=0.94, SMRM=0.04. |
|  | CO2 | 0.52-0.63 | 0.809 |  |
|  | CO3 | 0.48-0.58 | 0.722 |  |
|  | CO4 | 0.56-0.60 | 0.798 |  |

^a^ Spearman correlation by SPSS.20

^b^ Exploratory Factor Analysis (1 fixed factor) with Principal Axis Factoring estimation method by SPSS.20.

^C^ Weighted Least Squares estimation method was used for confirmatory factor analysis using LISREL version 9.1. NFI: Normed Fit Index; ≥0.9 indicating good fit. CFI: Comparative Fit Index; ≥0.9 indicating good fit. SRMR: Standardised Root Mean Square Residual; ≤0.05 indicating acceptable fit.
